# Efficient Aggregation-Induced Delayed Fluorescence Luminogens for Solution-Processed OLEDs With Small Efficiency Roll-Off

**DOI:** 10.3389/fchem.2020.00193

**Published:** 2020-04-07

**Authors:** Zheyi Cai, Hao Chen, Jingjing Guo, Zujin Zhao, Ben Zhong Tang

**Affiliations:** ^1^State Key Laboratory of Luminescent Materials and Devices, Guangdong Provincial Key Laboratory of Luminescence From Molecular Aggregates, Guangzhou, China; ^2^Department of Chemistry, Hong Kong Branch of Chinese National Engineering Research Center for Tissue Restoration and Reconstruction, The Hong Kong University of Science and Technology, Kowloon, China

**Keywords:** aggregation-induced delayed fluorescence, thermally activated delayed fluorescence, electroluminescence, organic light-emitting diodes, efficiency roll-off

## Abstract

Purely organic small molecules with thermally-activated delayed fluorescence have a high potential for application in organic light-emitting diodes (OLEDs), but overcoming severe efficiency roll-off at high voltages still remains challenging. In this work, we design and synthesize two new emitters consisting of electron-withdrawing benzoyl and electron-donating phenoxazine and 9,9-dihexylfluorene. Their electronic structures, thermal stability, electrochemical behaviors, photoluminescence property, and electroluminescence performance are thoroughly investigated. These new emitters show weak fluorescence in dilute solution, but they can emit strongly with prominent delayed fluorescence in the aggregated state, indicating the aggregation-induced delayed fluorescence (AIDF) character. The solution-processed OLEDs based on the two emitters show high external quantum efficiency of 14.69%, and the vacuum-deposited OLEDs can also provide comparable external quantum efficiency of 14.86%. Significantly, roll-offs of the external quantum efficiencies are very small (down to 0.2% at 1,000 cd m^−2^) for these devices, demonstrating the evidently advanced efficiency stability. These results prove that the purely organic emitters with AIDF properties can be promising to fabricate high-performance solution-processed OLEDs.

## Introduction

Organic light-emitting diodes (OLEDs) are attracting considerable attention across academia and industry because of their advantages of flexibility, fast response, high stability, light weight, and so forth. Generally, for conventional fluorescent organic materials, the ratio of electrically generated singlet and triplet excitons is 1:3, leading to a low internal quantum efficiency (IQE) limited to only 25%. One way to enhance the IQE is making full use of triplet excitons. As for phosphorescent materials, the theoretical maximum value of the IQE can reach 100%, resulting from utilizing both triplet and singlet excitons, but most of these materials have to incorporate precious heavy metals to promote intersystem crossing (ISC). The OLEDs based on such materials have high costs in noble metals, and often encounter aggregation or concentration caused emission quenching. Another way to enhance the IQE of purely organic materials is transforming triplet excitons to singlet excitons. According to the reports in recent years, there are several strategies to make use of the non-radiative triplet excitons, and thermally activated delayed fluorescence (TADF) is the method with most potential, in which triplet excitons can be converted to singlet excitons via reverse intersystem crossing (RISC) due to small singlet–triplet energy gap (Δ*E*_ST_) (Gong et al., 2011; Uoyama et al., 2012; Hirata et al., 2015; Kang et al., 2018). TADF materials are able to harvest both singlet and triplet excitons and thus can reach high exciton utilization without noble metal, but suffer from severe efficiency roll-off (Rajamalli et al., 2016, 2017; Xie et al., 2017). Recently, by taking the advantages of aggregation-induced emission (AIE) and TADF, a new molecular design strategy of aggregation-induced delayed fluorescence (AIDF) was proposed and a series of novel luminogens based on AIDF were developed. These AIDF materials could not only harness both singlet and triplet excitons, but also showed the merit of very small efficiency roll-off at high luminance (Huang et al., 2017; Guo et al., 2018, 2019).

On the other hand, compared to vacuum deposition, solution-processed film preparation techniques, including spin-coating, inkjet printing, roll to roll processing, etc., are fitter to manufacture large-area OLED devices with lower cost and less material waste (Gather et al., 2011; Gong et al., 2013; Cho et al., 2014; Albrecht et al., 2015; Zeng et al., 2019). Currently, luminescent polymers are the major choice to fabricate solution-processed devices due to their excellent film-forming ability (Lee et al., 2016; Shao et al., 2017; Kim et al., 2018; Zou et al., 2018). However, it is generally hard to remove the metal catalyst residue completely from the products, and as a result they are in low purity and have defects in many cases, which undermine their EL performance. Meanwhile, the reproducibility is another problem for the polymers. In opposition, small molecules have the advantages of clearly defined structures, easy purification, and better photoluminescence (PL) performance. So, in addition to conventional fluorescent and phosphorescent small molecules (Zhao et al., 2007, 2009; Yang et al., 2018), developing solution-processable small molecules with delayed fluorescence is of high significance. However, the currently reported solution-processable TADF molecules also suffer from severe efficiency roll-off at high voltages (Wu et al., 2009; Suzuki et al., 2015; Zhong et al., 2020). To solve this problem, in this work, we designed and synthesized two small molecules with AIDF property. Long alkyl chains are introduced into the molecules to enhance the film-forming ability for solution-processed OLED devices. They emit strong yellow to orange-yellow light with evident delayed fluorescence in solid film. The solution-processed OLEDs using them as emitting layers exhibit high EL efficiencies and very small efficiency roll-off.

## Experimental

### Synthesis

#### 9,9-Dihexyl-9H-fluorene (1)

Potassium *tert*-butoxide (16.83 g, 150 mmol) was added to a mixture of fluorene (8.30 g, 50 mmol) and 1-bromohexane (17.45 mL, 125 mmol) in dehydrated tetrahydrofuran (100 mL) and stirred for 12 h under 65°C. The reaction mixture was poured into water and extracted with dichloromethane several times. The combined organic layers were washed with water twice, and then dried over anhydrous NaSO_4_. After filtration, the crude product was concentrated and purified by column chromatography on silica gel (petroleum ether) to afford **1** as colorless liquid in 97% yield (16.21 g). ^1^H NMR (500 MHz, CDCl_3_) δ (TMS, ppm): 7.70–7.65 (m, 2H), 7.33–7.23 (m, 6H), 1.99–1.92 (m, 4H), 1.13–0.98 (m, 12H), 0.74 (t, *J* = 7.2 Hz, 6H), 0.67–0.54 (m, 4H). ^13^C NMR (125 MHz, CDCl_3_) δ (TMS, ppm): 150.65, 141.12, 126.97, 126.76, 122.79, 119.62, 55.12, 39.69, 32.17, 29.75, 23.57, 22.49, 13.99.

#### (9,9-Dihexyl-9H-fluoren-2-yl)(4-fluorophenyl) Methanone (2a) and (9,9-dihexyl-9H-fluorene-2,7-diyl) bis(4-fluorophenyl)Methanone (2b)

Aluminum trichloride (12.00 g, 90 mmol) was added into a stirred solution of **1** (9.70 g, 29 mmol) and 4-fluorobenzoyl chloride (14.22 g, 90 mmol) in dehydrated dichloromethane (50 mL) in 45°C and stirred for 6 h. The reaction was quenched with ice water and hydrochloric acid (50 mL, 2:1 v/v), and extracted with dichloromethane several times. The combined organic layers were washed with water twice, and then dried over anhydrous NaSO_4_. After filtration and solvent evaporation under reduced pressure, the residue was purified by column chromatography on silica gel (dichloromethane: petroleum ether, 2:3 v/v) to afford **2a** as yellow solid in 50% yield (6.61 g) and **2b** as yellow solid in 18% yield (3.02 g). For **2a**, ^1^H NMR (500 MHz, CD_2_Cl_2_) δ (TMS, ppm): 7.83–7.75 (m, 4H), 7.71–7.64 (m, 4H), 7.44–7.32 (m, 3H), 2.05–1.97 (m, 4H), 1.16–0.98 (m, 12H), 0.76 (t, *J* = 7.2 Hz, 6H), 0.66–0.56 (m, 4H). ^13^C NMR (125 MHz, CD_2_Cl_2_) δ (TMS, ppm): 196.66, 153.28, 152.12, 147.08, 141.11, 138.51, 136.91, 132.80, 132.77, 130.91, 129.74, 128.35, 128.19, 125.84, 124.46, 121.96, 120.65, 56.63, 41.38, 32.81, 30.90, 25.10, 23.84, 15.05, 0.90. For **2b**, ^1^H NMR (500 MHz, CDCl_3_) δ (TMS, ppm): 7.91–7.84 (m, 6H), 7.83–7.79 (m, 4H), 7.23–7.16 (m, 4H), 2.06–1.99 (m, 4H), 1.17–1.01 (m, 12H), 0.78 (t, *J* = 7.2 Hz, 6H), 0.70–0.60 (m, 4H). ^13^C NMR (125 MHz, CDCl_3_) δ (TMS, ppm): 198.49, 168.36, 164.38, 151.96, 144.94, 137.63, 134.25, 132.74, 129.80, 125.55, 119.88, 114.74, 113.69, 57.91, 39.34, 32.03, 29.55, 23.93, 22.55, 15.40.

#### ((4-(10H-Phenoxazin-10-yl)phenyl)(9,9-dihexyl-9H-fluoren-2-yl)methanone)) (FC6-BP-PXZ)

A mixture of **2a** (0.46 g, 1.0 mmol), phenoxazine (0.24 g, 1.3 mmol) and potassium *tert*-butoxide (0.23 g, 2.0 mmol) in deaerated *N, N*-dimethylformamide (20 mL) was heated up to 130°C and stirred for 12 h under nitrogen. After cooling down to room temperature, the reaction was quenched with water (20 mL), and extracted with dichloromethane several times. The combined organic layers were washed with water twice, and then dried over anhydrous NaSO_4_. After filtration and solvent evaporation under reduced pressure, the residue was purified by column chromatography on silica gel (dichloromethane: petroleum ether, 1:1 v/v) to afford orange solid of FC6-BP-PXZ in 37% yield (0.23 g). ^1^H NMR (500 MHz, CD_2_Cl_2_) δ (TMS, ppm): 8.05–8.02 (m, 2H), 7.90 (s, 1H), 7.86–7.79 (m, 3H), 7.54–7.49 (m, 2H), 7.45–7.35 (m, 3H), 6.94–6.42 (m, 6H), 6.06 (s, 2H), 2.14–1.97 (m, 4H), 1.18–0.97 (m, 12H), 0.75 (t, *J* = 7.1 Hz, 6H), 0.70–0.57 (m, 4H). ^13^C NMR (125 MHz, CD_2_Cl_2_) δ (TMS, ppm): 195.86, 153.14, 151.38, 146.89, 140.24, 139.06, 136.09, 133.05, 130.32, 127.48, 124.87, 123.61, 120.45, 119.25, 113.41, 56.84, 40.52, 31.93, 29.54, 24.25, 22.48, 15.73. HRMS (C_44_H_45_NO_2_): *m/z* 619.1476 [M^+^, calcd 619.3450].

#### ((9,9-Dihexyl-9H-fluorene-2,7-diyl)bis((4-(10H-phenoxazin-10-yl)phenyl)methanone)) (FC6-2BP-PXZ)

A mixture of **3b** (0.8674 g, 1.5 mmol), phenoxazine (0.8238 g, 4.5 mmol) and potassium *tert*-butoxide (0.5049 g, 4.5 mmol) in deaerated *N, N*-dimethylformamide (20 mL) was heated up to 130°C and stirred for 12 h under nitrogen. After cooling down to room temperature, the reaction was quenched with water (20 mL), and extracted with dichloromethane several times. The combined organic layers were washed with water twice, and then dried over anhydrous NaSO_4_. After filtration and solvent evaporation under reduced pressure, the residue was purified by column chromatography on silica gel (dichloromethane: petroleum ether, 1:1 v/v) to afford orange solid of FC6-2BP-PXZ in 66% yield (0.90 g). ^1^H NMR (500 MHz, CD_2_Cl_2_) ^1^H NMR (500 MHz, CD_2_Cl_2_) δ (TMS, ppm): 8.09–8.03 (m, 4H), 7.99–7.94 (m, 4H), 7.93–7.88 (m, 2H), 7.56–7.42 (m, 4H), 6.84–6.56 (m, 12H), 6.07 (s, 4H), 2.16–2.08 (m, 4H), 1.18–0.98 (m, 12H), 0.74 (t, *J* = 7.0 Hz, 6H), 0.72–0.65 (m, 4H). ^13^C NMR (125 MHz, CDCl_3_) δ (TMS, ppm): 195.56, 137.77, 136.80, 133.74, 130.82, 130.12, 123.29, 120.45, 115.75, 31.49, 23.98, 22.54, 13.97. HRMS (C_63_H_56_N_2_O_2_): *m/z* 904.1583 [M^+^, calcd 904.4240].

### OLED Fabrication and Characterization

The solution-processed devices were fabricated on clean glass substrates pre-coated with a 180 nm-thin layer of indium tin oxide (ITO) with a sheet resistance of 10 Ω per square. The ITO surface was treated with an ultrasonic detergent bath for 90 min, followed by soaking in ultrasonic de-ionized water for 20 min, then dried at 120°C for 1 h, and UV/Ozone cleaning for 15 min before spin-coating. A 50 nm-thin poly(3,4-ethylenedioxythiophene):poly(styrenesulfonate) (PEDOT:PSS) layer was spin-coated onto ITO surface at 3,000 rpm, then baked at 150 °C for 50 min to remove the residual water. Then, the substrates were moved into a glovebox under a nitrogen atmosphere, and a poly(9-vinylcarbazole) (PVK) layer was spin-coated onto the PEDOT:PSS layer at 2,500 rpm (the thickness achieved 30 nm) from a filtered 10 mg mL^−1^ chlorobenzene solution, followed by drying at 120°C for 20 min. Then, the emitting layer was spin-coated according to the configuration requirement. Solutions of FC6-BP-PXZ (30 wt%) or FC6-2BP-PXZ (10 wt%) doped in 4,4'-bis(carbazol-9-yl)biphenyl (CBP) with an overall concentration of 20 mg mL^−1^ in toluene were spin-coated at 2,500 rpm for 45 s to get films with a thickness of 50 nm. Finally, an electron-transport layer of 1,3,5-tri(*m*-pyrid-3-yl-phenyl)benzene (TmPyPB), a LiF layer, and an Al layer were deposited consecutively onto the spin-coated film in a vacuum chamber under 10^−4^ Pa. Vacuum-evaporation OLEDs were fabricated on clean glass substrates pre-coated with a 180-nm-thin layer of ITO with a sheet resistance of 10 Ω per square. Organic layers were deposited by high-vacuum (5 × 10^−4^ Pa) thermal evaporation onto a glass substrate pre-coated with an ITO layer. All organic layers were deposited sequentially. Thermal deposition rates for the organic materials, LiF and Al were 0.5, 0.5, and 1 Å s^−1^, respectively. The thicknesses of the vacuum deposited layers were monitored by quartz crystal microbalance and were calibrated by Dektak XT profilometer. The emission area of the devices is 3 × 3 mm^2^ as shaped by the overlapping area of the anode and cathode. All the device characterization steps were carried out at room temperature under ambient laboratory conditions without encapsulation. EL spectra were taken by an optical analyzer, FlAME-S-VIS-NIR. Current density and luminance vs. driving voltage characteristics were measured by Keithley 2400 and Konica Minolta chromameter CS-200. External quantum efficiencies were calculated by assuming that the devices were Lambertian light sources.

## Results and Discussion

### Synthesis and Thermal Stability

**Scheme 1:**
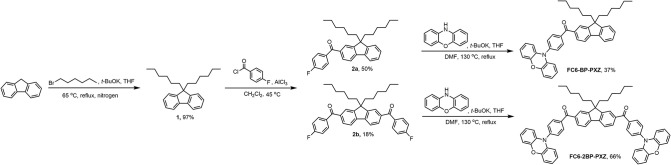
Synthetic routes of FC6-BP-PXZ and FC6-2BP-PXZ.

The target compounds FC6-BP-PXZ and FC6-2BP-PXZ were simply and efficiently synthesized. As shown in Scheme 1, 9,9-dihexyl-9H-fluorene (**1**) that was prepared by the method in reported literature underwent Friedel-Crafts acylation reaction with compound **2** to yield intermediate **3a** and **3b**. The treatments of **3a** and **3b** with phenoxazine (PXZ) furnished the final compounds FC6-BP-PXZ and FC6-2BP-PXZ, respectively, in high yields. The molecular structures had been well-characterized by NMR and high-resolution mass spectra. Owing to the presence of hexyl groups, both compounds have good solubility in common organic solvents, such as chlorobenzene, toluene, chloroform, dichloromethane, tetrahydrofuran (THF), and so on, but do not dissolve easily in water because of the hydrophobic structures. The thermal stability of both compounds is characterized thoroughly by thermogravimetric analysis (TGA) and differential scanning calorimetry (DSC) under nitrogen. FC6-BP-PXZ and FC6-2BP-PXZ show high decomposition temperatures of 340.1 and 424.5°C, and high glass-transition temperatures of 85.7 and 84.0°C, respectively (Figure S1). The good thermal and morphological stabilities of both compounds enable them to function in OLEDs, and benefit device performances.

### Photophysical Behavior

**Figure 1:**
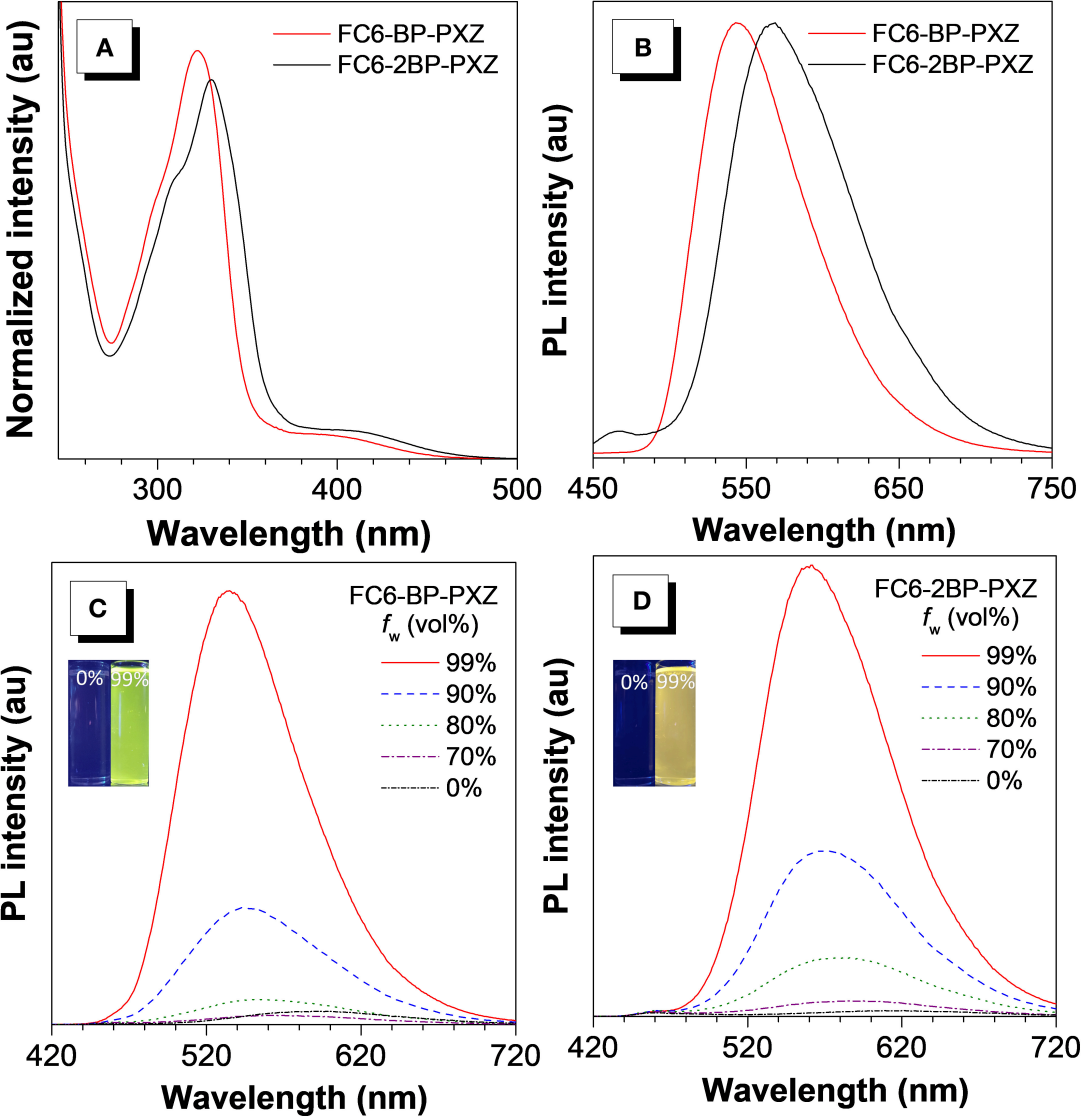
**(A)** Absorption spectra in THF solutions (10^−*5*^ M) and **(B)** PL spectra in neat films of FC6-BP-PXZ and FC6-2BP-PXZ. PL spectra of **(C)** FC6-BP-PXZ and **(D)** FC6-2BP-PXZ in THF-water mixtures with different water fractions (*f*_w_).

**Figure 2:**
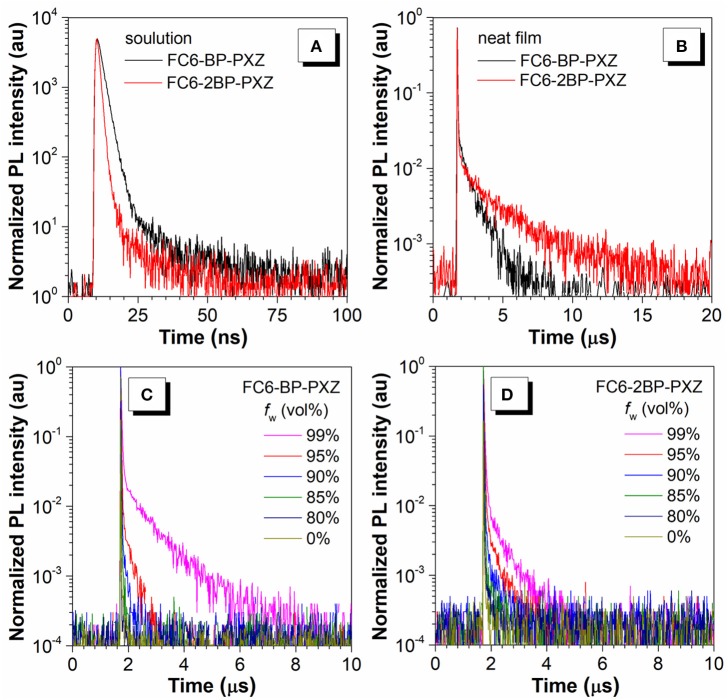
Transient PL decay spectra of FC6-BP-PXZ and FC6-2BP-PXZ **(A)** in THF solutions (10^−*5*^ M) and **(B)** in neat films, measured at 300 K under nitrogen. The water fraction dependent transient PL decay spectra of **(C)** FC6-BP-PXZ and **(D)** FC6-2BP-PXZ in THF-water solutions.

FC6-BP-PXZ and FC6-2BP-PXZ show strong absorption bands at around 317 and 330 nm in THF solution, associated with π-π^*^ transition of the molecules. There are also weak absorption bands at around 400 nm resulting from twisted intramolecular charge transfer (TICT) from the electronic donating-accepting (D-A) structure (Figure 1A) (Kashihara et al., 2017; Thorat et al., 2017; Higginbotham et al., 2018). In THF solution, FC6-BP-PXZ and FC6-2BP-PXZ emit weakly at 543 and 550 nm, with low fluorescence quantum yields (Φ_F_s) of 3.5 and 2.0%, (Figure 2A) respectively. However, they can emit strongly at 544 and 567 nm with higher Φ_F_s of 32.0 and 17.0% in neat films at 300 K, respectively, indicating they have AIE property (Figures 1B, 2B and Table S2). To further confirm this, the PL behaviors are measured in their water-THF mixtures (Figures 1C,D). The emission intensity is much stronger and the emission peak is blue-shifted when water fraction in the mixture gets high. Since these compounds are insoluble in water, they are prone to form aggregates when the water fraction becomes high, indicating the enhanced emission is caused by the aggregate formation. In the aggregated state, the intramolecular motions that are active in solution state are restricted by the spatial constraint. In consequence, the non-radiative decay channel is blocked, and the excited state energy can be released as photons, leading to greatly enhanced emissions (Mei et al., 2015; Zhao et al., 2015; Shen et al., 2018). These results confirm that both compounds indeed have AIE properties. And from the fluorescence and phosphorescence spectra of FC6-BP-PXZ and FC6-2BP-PXZ at 77 K (Figure S2), the Δ*E*_ST_ values are estimated to be 0.017 and 0.068 eV, respectively, which are small enough for promoting RISC process.

The transient PL decay spectra show that both FC6-BP-PXZ and FC6-2BP-PXZ have short average lifetimes within 2.0 ns and the delayed fluorescence is hardly observed in solution. However, FC6-BP-PXZ and FC6-2BP-PXZ in neat films show much longer mean lifetimes of 0.22 and 0.62 μs, with prompt components of 20.7 ns and 29.0 ns and evident delayed components of 0.66 and 2.12 μs (Figures 2C,D), respectively. Moreover, the transient PL decay spectra in water-THF mixtures are further measured. It can be seen that when the water fraction increases, the mean lifetimes become longer. And the ratios of delayed components and the rate constant of RISCs are enhanced greatly (Table 1, Table S1). These finding demonstrate that the delayed fluorescence of both compounds is induced by the aggregation formation, indicative of their AIDF nature apparently. In solution state, the excited state is readily deactivated by fast internal conversion (IC) of vigorous intramolecular motions, and thus the ISC and RISC processes cannot readily occur. In the aggregated state, however, the intramolecular motions are greatly suppressed by spatial hindrance, and the IC channel is blocked. Therefore, given their small Δ*E*_ST_ values, the ISC and RISC are able to occur, leading to noticeable delayed fluorescence (Guo et al., 2018, 2019).

**Table 1:** Photophysical properties of FC6-BP-PXZ and FC6-2BP-PXZ.

	**Solution^a^**			**Neat film^b^**				
	**λ_abs_(nm)**	**λ_em_(nm)**	**Φ_F_^c^ (%)**	***λ_*em*_* (nm)**	**Φ_F_^c^ (%)**	***τ_prompt_*^d^ (ns)**	***τ_delayed_*^d^ (μs)**	**Δ*E*_ST_^e^(eV)**
FC6-BP-PXZ	317	543	3.5	544	32.0	20.7	0.7	0.017
FC6-2BP-PXZ	330	550	2.0	567	17.0	29.0	2.1	0.068

a *In THF solution (10^−5^ M) at room temperature*.

b *Spin-coated on a quartz substrate*.

c *Absolute fluorescence quantum yield determined by a calibrated integrating sphere under nitrogen at room temperature*.

d *PL lifetimes of prompt (τprompt) and delayed (τdelayed) decay components evaluated at 300 K under vacuum*.

e *Estimated from the high-energy onsets of fluorescence and phosphorescence spectra at 77 K*.

### Theoretical Calculation

**Figure 3:**
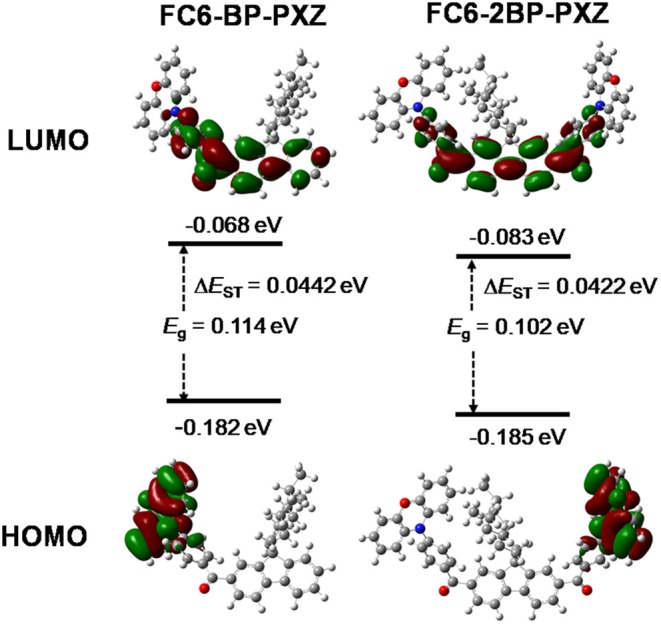
Optimized molecular structures and frontier orbital amplitude plots of FC6-BP-PXZ and FC6-2BP-PXZ, calculated by PBE0 hybrid functional at the basis set level of 6-31G^*^.

The DFT/TDDFT calculation is applied to investigate the molecular orbital amplitude plots and energy levels of the highest occupied molecular orbitals (HOMOs) and lowest unoccupied molecular orbitals (LUMOs) of both compounds. As shown in Figure 3, the HOMOs of both compounds are distributed on fluorene and benzoyl moieties, and the LUMOs are concentrated on PXZ. The apparently separated distribution of HOMOs and LUMOs is necessary to achieve small Δ*E*_ST_ values and thus to promote RISC process and delayed fluorescence. The theoretical Δ*E*_ST_ values of FC6-BP-PXZ and FC6-2BP-PXZ are calculated to as small as 0.0442 and 0.0422 eV, respectively.

### Electrochemical Property

**Figure 4:**
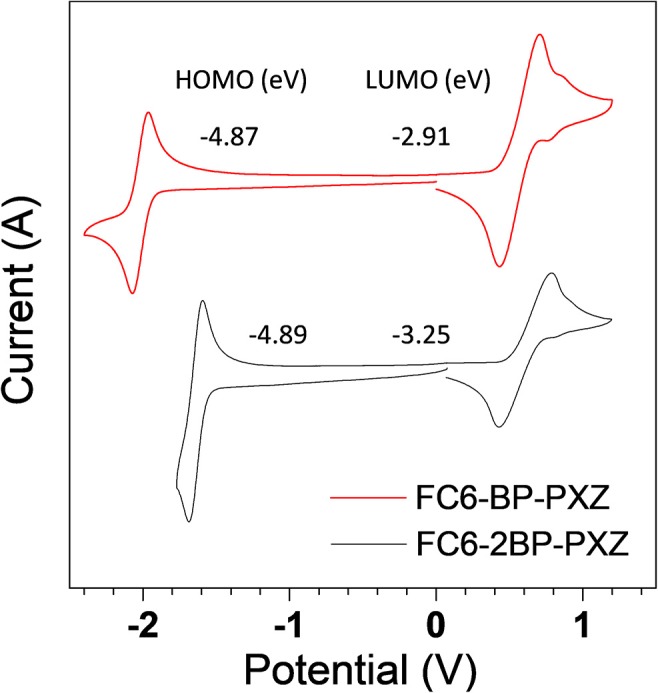
Cyclic voltammograms of FC6-BP-PXZ and FC6-2BP-PXZ measured in acetonitrile containing 0.1 M tetra-*n*-butylammonium hexafluorophosphate. Scan rate: 100 mV s^−1^.

Cyclic voltammetry (CV) is conducted to investigate the electrochemical behaviors of FC6-BP-PXZ and FC6-2BP-PXZ in a solution of acetonitrile with tetra-*n*-butylammonium hexafluorophosphate (Bu_4_NPF_6_, 0.1 M). Three-electrode system (Ag/Ag^+^, platinum wire and glassy carbon electrodes as reference, counter and work electrodes, respectively) is used and the scan rate is 100 mV s^−1^ in the measurement. As illustrated in Figure 4, both compounds undergo reversible oxidation and reduction processes, indicating good electrochemical stability. The oxidation peaks of FC6-BP-PXZ and FC6-2BP-PXZ are both located at 0.435 V and the reduction peaks at −1.96 and −1.60 V, respectively. The HOMO energy levels of FC6-BP-PXZ and FC6-2BP-PXZ are calculated to be −4.87 to −4.89 eV, respectively, from the onset oxidation potentials, and the LUMO energy levels are −2.91 to −3.25 eV, from the onset reduction potentials (HOMO = –[*E*_ox_ + 4.8] eV, and LUMO = –[*E*_re_ + 4.8] eV, in which *E*_ox_ and *E*_re_ represent the onset oxidation and reduction potentials relative to Fc/Fc^+^, respectively).

### Electroluminescence

**Figure 5:**
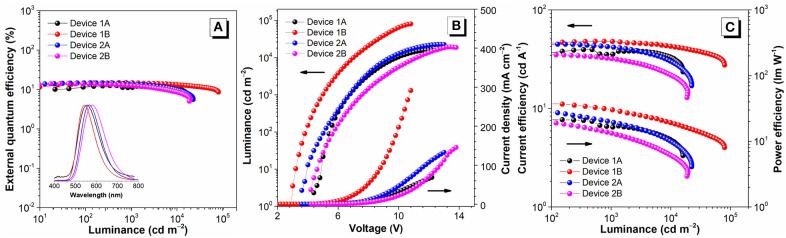
**(A)** Luminance–external quantum efficiency with EL spectra, **(B)** luminance–voltage–current density, and **(C)** current efficiency–luminance–power efficiency characteristics of the devices. Inset in **(A)**: photographs of solution-processed devices (left: device 1A; right: device 2A).

Based on the excellent PL properties of FC6-BP-PXZ and FC6-2BP-PXZ, their EL performances in solution-processed OLEDs and vacuum-deposited OLEDs were further investigated. The key data of these OLEDs are summarized in Table 2, and the relative characteristic curves are plotted in Figure 5. The solution-processed OLEDs with a configuration of ITO/PEDOT:PSS (50 nm)/PVK (30 nm)/emitter/TmPyPB (40 nm)/LiF (1 nm)/Al [Device 1A: emitter = CBP:30 wt% FC6-BP-PXZ (50 nm); Device 2A: emitter = CBP:10 wt% FC6-2BP-PXZ (50 nm)] were fabricated, in which PEDOT:PSS and LiF were used as hole- and electron-injecting layers, respectively; PVK and TmPyPB were selected as hole- and electron-transporting layers, respectively; CBP functioned as a host. As shown in Figure 5, the turn-on voltage at 10 cd m^−2^ of Devices 1A and 2A are 5.0 and 3.9 V, radiating orange-yellow light at ~555 nm (CIE_x,y_ = 0.402, 0.549) and ~568 nm (CIE_x,y_ = 0.432, 0.543), respectively. The maximum luminance (*L*_max_), current efficiency (η_C,max_), power efficiency (η_P,max_) and external quantum efficiency (η_ext,max_) of Device 2A are 22530 cd m^−2^, 44.83 cd A^−1^, 32.03 lm W^−1^, and 14.69%, respectively. It is significant that the external quantum efficiency at luminance of 1,000 cd m^−2^ is 13.80%, showing a very low efficiency roll-off of 6.06%. The EL properties of Device 1A is somewhat inferior than those of Device 2A.

**Table 2:** EL performances of OLEDs based on FC6-BP-PXZ and FC6-2BP-PXZ^a^.

		**V_on_ (V)**	**Maximum values**					**Values at 1,000 cd m^−2^**				
			**η_C_ (cd A^–1^)**	**η_P_ (lm W^–1^)**	**η_ext_ (%)**	**L (cd m^–2^)**	**η_C_ (cd A^–1^)**	**η_P_ (lm W^–1^)**	**η_ext_(%)**	**RO (%)**	**λ_EL_ (nm)**	**CIE (x, y)**
FC6-BP-PXZ	1A	5.0	39.61	21.29	12.49	16100	35.49	16.39	11.20	10.30	555	(0.402, 0.549)
	1B	3.2	48.02	38.91	14.86	80507	47.84	30.06	14.83	0.20	544	(0.392, 0.569)
FC6-2BP-PXZ	2A	3.9	44.83	32.03	14.69	22530	41.76	19.28	13.80	6.06	568	(0.432, 0.543)
	2B	4.6	36.12	25.52	14.12	19455	34.10	31.98	13.22	6.33	582	(0.446, 0.523

a *V_on_ = turn-on voltage at 10 cd m^−2^*;

*η_c_ = current efficiency; η_p_ = power efficiency; η_ext_ = external quantum efficiency; RO = current efficiency roll-off from maximum value to that at 1,000 cd m^−2^; λ_EL_ = electroluminescence maximum; CIE = Commission Internationale de I'Eclairage coordinates at condition of maximum η_ext_*.

To further investigate the EL properties of both compounds, vacuum-deposited OLEDs with a configuration of ITO/TAPC (25 nm)/emitter/TmPyPB (55 nm)/LiF (1 nm)/Al [Device 1B: emitter = CBP:30 wt% FC6-BP-PXZ (35 nm); Device 2B: emitter = CBP: 10 wt% FC6-2BP-PXZ (35 nm)] were fabricated, in which 4,4'-cyclohexylidenebis[*N, N*-bis(p-tolyl)aniline] (TAPC) and TmPyPB were selected as hole- and electron-transporting layers, respectively. As shown in Figure 5, the turn-on voltage at 10 cd m^−2^ of Devices 1B and 2B are 3.2 V and 4.6 V, emitting orange-yellow light at ~544 nm (CIE_x,y_ = 0.392, 0.569) and ~582 nm (CIE_x,y_ = 0.446, 0.523), respectively. The *L*_max_, η_C,max_, η_P,max_, and η_ext,max_ of Devices 1B and 2B are 80,507 cd m^−2^, 48.02 cd A^−1^, 38.91 lm W^−1^ and 14.86%, and 19,455 cd m^−2^, 36.12 cd A^−1^, 25.52 lm W^−1^ and 14.12%, respectively, in which the efficiency roll-off is extremely small especially for Device 1B (0.20% at luminance of 1,000 cd m^−2^). These new emitters with AIDF property provide good EL performance, and according to the photophysical parameters, the exciton utilization of these OLEDs has approached nearly 100%. More importantly, extremely small efficiency roll-offs are successfully achieved, which should be an apparent advance to conventional TADF emitters for solution-processed OLEDs and vacuum-deposited OLEDs. And the AIDF character of the materials should be important for achieving high performance, which combines the superior features of efficient solid-state emission, high exciton utilization and low exciton quenching.

## Conclusions

In summary, two new emitters built with electron-withdrawing group benzoyl and electron-donating phenoxazine and 9,9-dihexylfluorene are designed and synthesized. They have high thermal and morphological stabilities and good electrochemical stability. Whereas, in dilute solution state they emit weakly with faint delayed fluorescence, they can emit strongly with prominent delayed fluorescence in the aggregated state, demonstrating the AIDF property. In addition, they fluoresce intensely in spin-coated films with notable delayed fluorescence, owing to the small Δ*E*_ST_, and thus fast RISC process. As a consequence, they can perform excellently as light-emitting layers in solution-processed OLEDs, providing high η_ext,max_ of up to 14.69% and very small efficiency roll-off at the luminance of 1,000 cd m^−2^, demonstrating the outstanding efficiency stability. On the other hand, their vacuum-deposited OLEDs also have good η_ext,max_ of up to 14.86% and negligible efficiency roll-off at 1,000 cd m^−2^. These results indicate the great potential of small molecules with AIDF property for the fabrication of high-performance solution-processed and vacuum-deposited OLEDs.

## Data Availability Statement

The datasets generated for this study are available on request to the corresponding author.

## Author Contributions

All authors listed have made a substantial, direct and intellectual contribution to the work, and approved it for publication.

### Conflict of Interest

The authors declare that the research was conducted in the absence of any commercial or financial relationships that could be construed as a potential conflict of interest.
