# Supplementary material for: Efficient Aggregation-Induced Delayed Fluorescence Luminogens for Solution-Processed OLEDs With Small Efficiency Roll-Off
Source: Front Chem. 2020 Apr 7;8:193. doi: 10.3389/fchem.2020.00193 (PMC7154159; doi:10.3389/fchem.2020.00193)
Supplement: Supplementary file 1 [file Data_Sheet_1.PDF]

Supplementary Material

Efficient aggregation-induced delayed fluorescence luminogens for solution-processed OLEDs with small efficiency roll-off

Zheyi Cai,[1] Hao Chen,[1] Jingjing Guo,[1] Zujin Zhao,*[1] and Ben Zhong Tang*[1,2]

[1] State Key Laboratory of Luminescent Materials and Devices, Guangdong Provincial Key Laboratory of Luminescence from Molecular Aggregates, South China University of Technology, Guangzhou 510640, China.

[2] Department of Chemistry, Hong Kong Branch of Chinese National Engineering Research Center for Tissue Restoration and Reconstruction, The Hong Kong University of Science and Technology, Clear Water Bay, Kowloon, Hong Kong, China.

Materials and Instruments

All the chemicals and reagents were purchased from commercial sources and used as received without further purification. ^{1}H and ^{13}C NMR spectra were measured on a Bruker AV 500 spectrometer at room temperature. High resolution mass spectra (HRMS) were recorded on a GCT premier CAB048 mass spectrometer operating in MALDI-TOF mode. UV-vis absorption spectra were measured on a Shimadzu UV-2600 spectrophotometer. PL spectra were recorded on a Horiba Fluoromax-4 spectrofluorometer. PL quantum yields were measured by using a Hamamatsu absolute PL quantum yield spectrometer C11347 Quantaurus_QY. The ground-state geometries were optimized using the density function theory (DFT) method with PBE0 hybrid functional at the basis set level of 6-31G*, and then the ΔE_{ST} values were calculated by time-dependent DFT (TDDFT) method at the same level. All the calculations were performed using Gaussian09 package.

Additional Spectra

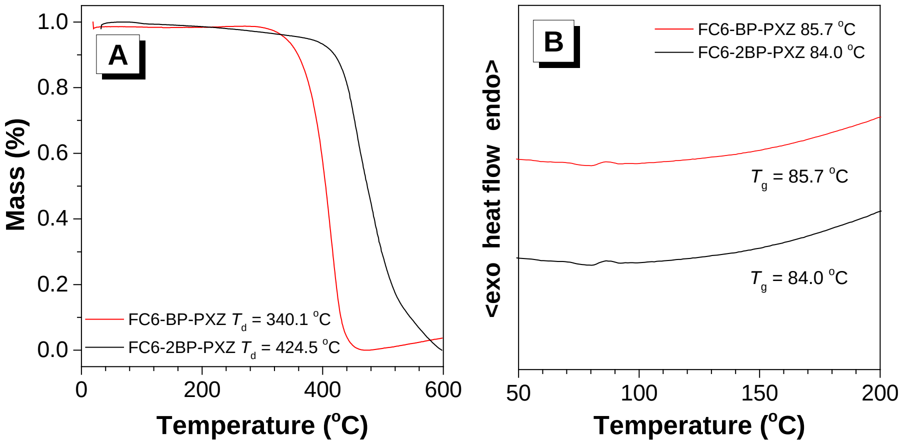

Fig. S1 (A) TGA and (B) DSC thermograms of FC6-BP-PXZ and FC6-2BP-PXZ, measured under nitrogen at a heating rate of 20 and 10 °C min^{-1}, respectively.

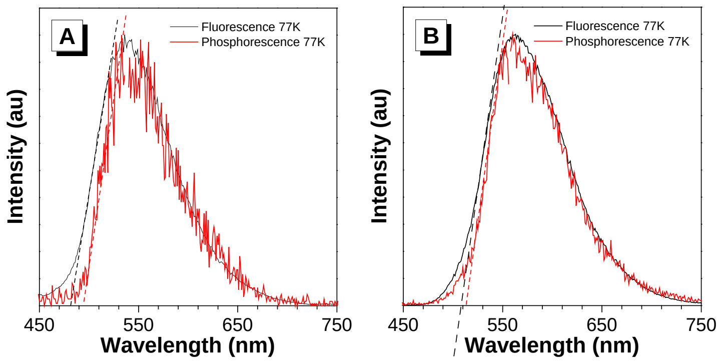

Fig. S2 Fluorescence and phosphorescence spectra of (A) FC6-BP-PXZ neat film and (B) FC6-2BP-PXZ neat film.

Estimation of Basic Photophysical Data

The quantum efficiencies and rate constants were determined using the following equations according to the following equations:

$\Phi_{prompt} = \Phi_F R_{prompt}$ (1)

$\Phi_{delayed} = \Phi_F R_{delayed}$ (2)

$k_F = \Phi_{prompt}/\tau_{prompt}$ (3)

$\Phi_F = k_F/(k_F + k_{IC})$ (4)

$\Phi_{prompt} = k_F/(k_F + k_{IC} + k_{ISC})$ (5)

$\Phi_{IC} = k_{IC}/(k_F + k_{IC} + k_{ISC})$ (6)

$\Phi_{ISC} = k_{ISC}/(k_F + k_{IC} + k_{ISC}) = 1 - \Phi_{prompt} - \Phi_{IC}$ (7)

$\Phi_{RISC} = \Phi_{delayed}/\Phi_{ISC}$ (8)

$k_{RISC} = (k_p k_d \Phi_{delayed})/(k_{ISC}\Phi_{prompt})$ (9)

$k_p = 1/\tau_{prompt}$; $k_d = 1/\tau_{delayed}$ (10)

Table S1. Photophysical data of neat films in FC6-2BP-PXZ and FC6-2BP-PXZ.[a]

	FC6-BP-PXZ	FC6-2BP-PXZ
	neat film	neat film
Φ_{F} (%)	32.0	17.0
τ_{prompt} (ns)	20.9	29.0
$\tau_{delayed}$ (ns)	661.3	2119.6
$R_{delayed}$ (%)	31.8	28.2
Φ_{prompt} (%)	21.8	12.2
$\Phi_{delayed}$ (%)	10.2	4.8
Φ_{ISC} (%)	31.8	28.2
Φ_{RISC} (%)	32.0	17.0
k_{F} ($\times 10^{6}$ s^{-1})	10.4	4.21
k_{IC} ($\times 10^{6}$ s^{-1})	22.2	20.5
k_{ISC} ($\times 10^{6}$ s^{-1})	15.2	9.73
k_{RISC} ($\times 10^{6}$ s^{-1})	2.2	0.6

[a] Abbreviations: Φ_{PL} = absolute photoluminescence quantum yield; τ_{prompt} and $\tau_{delayed}$ = lifetimes calculated from the prompt and delayed fluorescence decay, respectively; $R_{delayed}$ = the ratio of delayed components; Φ_{prompt} and $\Phi_{delayed}$ = quantum yields from prompt and delayed components, respectively, determined from the total Φ_{PL} and the proportion of the integrated area of each of the components in the transient spectra to the total integrated area; Φ_{ISC} = the intersystem crossing quantum yield; K_{F} = fluorescence decay rate; K_{IC} = internal conversion decay rate from S_1 to S_0; K_{ISC} = intersystem crossing decay rate from S_1 to T_1; K_{RISC} = the rate constant of reverse intersystem crossing process.

Table S2. Transient PL decay data of THF solutions and neat films of FC6-BP-PXZ and FC6-2BP-PXZ at 300 K under nitrogen.[a]

compound	state	$<\tau>$ (ns)	τ_1 (ns)	τ_2 (ns)	A_1	A_2	R_{prompt} (%)	$R_{delayed}$ (%)
FC6-BP-PXZ	THF solution	2.2	1.9	8.0	5694.1	56.94	–	–
	neat film	225.9	20.9	661.3	9336	138.1	68	32
FC6-2BP-PXZ	THF solution	1.0	0.2	0.9	2668.6	7274.4	–	–
	neat film	618.8	29.0	2119.6	10288.3	55.3	72	28

[a] The transient PL decay data were fitted by multiple-exponential function and the mean fluorescence lifetimes ($<\tau>$) were calculated by $<\tau> = \Sigma A_i\tau_i^2/\Sigma A_i\tau_i$, where A_i is the pre-exponential for lifetime τ_i. R_{prompt} and $R_{delayed}$ are individual component ratio for prompt and delayed fluorescence. $R_{prompt} = \tau_1A_1/(\tau_1A_1 + \tau_2A_2)$, $R_{delayed} = 1 - R_{prompt}$.
